# Platelet Count Predicts Adverse Clinical Outcomes After Ischemic Stroke or TIA: Subgroup Analysis of CNSR II

**DOI:** 10.3389/fneur.2019.00370

**Published:** 2019-04-12

**Authors:** Ming Yang, Yuesong Pan, Zixiao Li, Hongyi Yan, Xingquan Zhao, Liping Liu, Jing Jing, Xia Meng, Yilong Wang, Yongjun Wang

**Affiliations:** ^1^Department of Neurology, Beijing Tiantan Hospital, Capital Medical University, Fengtai District, China; ^2^China National Clinical Research Center for Neurological Diseases, Beijing, China; ^3^Center of Stroke, Beijing Institute for Brain Disorders, Beijing, China; ^4^Beijing Key Laboratory of Translational Medicine for Cerebrovascular Disease, Beijing, China

**Keywords:** ischemic stroke, platelet count, functional outcome, recurrent stroke, mortality

## Abstract

**Background:** The clinical significance of platelet count (PC) for ischemic cerebrovascular disease is not well-established and further risk stratification according to baseline PC within normal range has not been reported before. We aim to evaluate the prognostic effect of baseline circulating PC within normal range on the risk of long-term recurrent stroke, mortality and functional outcomes after ischemic stroke or TIA.

**Methods:** We derived data from eligible patients with ischemic stroke or TIA from the China National Stroke Registry (CNSR) II. Participants were divided into quintiles according to baseline PC within normal range (100–450 × 10^9^/L). Multivariable cox regression and logistic regression were adopted to explore the correlation of baseline PC with recurrent stroke, mortality and poor functional outcomes (modified Rankin Scale 3~6) within 1-year follow-up.

**Results:** Among the16842 eligible participants, the average age was 64.7 ± 11.9, 1,241 (7.4%) had recurrent stroke, 1,377 (8.2%) died, and 3,557 (21.1%) ended up with poor functional outcomes after 1-year follow-up. Compared with the third PC quintile (186–212 × 10^9^/L), patients in the top quintile (249–450 × 10^9^/L) presented with increased risk of recurrent stroke (adjusted hazard ratio 1.21, [1.02–1.45]), all-cause mortality (adjusted hazard ratio 1.43, [1.19–1.73]), and poor functional outcome (adjusted odds ratio 1.49, [1.28–1.74]), while patients in the lowest PC quintile(100–155 × 10^9^/L) had higher risk of poor functional outcome (adjusted odds ratio 1.19, [1.02–1.38]).

**Conclusion:** In ischemic stroke or TIA patients with platelet count within normal range, platelet count may be a qualified predictor for long-term recurrent stroke, mortality, and poor functional outcome.

## Introduction

Platelets exert a critical role in the pathogenesis of atherosclerotic complications of cardio-cerebrovascular disease, contributing to thrombus formation, and embolism (1, 2). Previous literature reported that platelets of various size and density are produced by megakaryocytes of different size and stages of maturation in different clinical conditions, suggesting various platelet patterns in different clinical conditions (3). Abnormal PC has been confirmed as a readily available biomarker of poor prognosis in cardiovascular disease patients (4), critically ill patients (5), and general population (6). However, the clinical significance of PC, especially for ischemic cerebrovascular disease, is not well-established and further risk stratification according to baseline PC within normal range for ischemic stroke or TIA patients has not been reported before.

These preliminary findings and the important role of platelets in the pathophysiology of atherosclerosis probably qualify platelet count an easily available and novel prognostic biomarker for ischemic cerebrovascular disease, though not as definitive conclusions. The hypothesis of the present study was that ischemic stroke or TIA patients with PC differing from the general population median had increased stroke recurrence or risk of poor clinical outcome. This study primarily aimed to explore the prognostic significance of baseline PC within normal range on long-term follow-up clinical outcomes for ischemic stroke or TIA patients through a nationwide prospective registry study.

## Methods

### Study Population

We derived data from the China National Stroke Registry II (CSNR II) (7), a nationwide, multicenter, prospective registry study launched in 2012 in China aiming to evaluating stroke care delivery in clinical practice. A total of 25,018 patients were included in the CNSR II that met the following criteria: age ≥18 years; diagnosis within 7 days of the index event of ischemic stroke, transient ischemic attack (TIA), spontaneous intracerebral hemorrhage (ICH), or subarachnoid hemorrhage (SAH) confirmed by brain imaging; or direct hospital admission from clinic or emergency department.

Among all the enrolled patients in the registry, 21,592 were diagnosed with ischemic stroke or TIA and therein 16,842 patients with PC within a priori defined range of 100–400 × 10^9^/L were involved in the current analysis after excluding patients with pregnancy/within post-partum 6 weeks (*n* = 3), sickle-cell disease (*n* = 1), thrombocytopenia (8) (PC < 100 × 10^9^/L) (*n* = 542), and thrombocytosis (9) (PC > 450 × 10^9^/L) (*n* = 33), bleeding tendency/history caused by blood platelet disorders or coagulation factor deficiency (*n* = 114), missing PC (*n* = 1,703), and lost to 1 year follow-up (*n* = 2,354).

Baseline information on demographics, risk factors, pre-hospital medication history, important laboratory data (alanine aminotransferase, white blood cells, CRP, hsCRP, PC, etc.), treatment regimens, and complications were collected through face-to-face interviews by trained coordinators (neurologists from participating hospitals) (7). Data collection and the protocol of the CNSR II study were approved by the Central Institutional Review Board of the Beijing Tiantan Hospital, and all written informed consents were acquired from patients or their legal proxies.

### Platelet Count Testing

Fasting whole blood samples from venipuncture were taken within 24 h of admission into a vacutainer tube containing EDTA, and kept at room temperature. Afterwards, PC was analyzed by automated hematology analyzer within 1 h after sample collection at each research center. All measurements were performed by laboratory personnel blinded to subjects' clinical situations. PC as a continuous variable was divided into quintiles in accordance with previous studies (4, 6).

### Outcome Assessment

Patients enrolled were followed up for 1 year by telephone interview according to the protocol of CSNR II study. Clinical endpoints, including recurrent stroke, all-cause mortality, and modified Rankin Scale(mRS) score (ranging from 0 to 6), were collected by trained research coordinators who were blinded to subjects' baseline characteristics. Adverse clinical outcomes were defined as recurrent stroke, all-cause mortality, and poor functional outcome (mRS 3~6). Recurrent stroke included both ischemic and hemorrhagic stroke during follow-up period.

### Statistical Analysis

Continuous variables are described as mean ± SD or median with interquartile range and categorical variables as proportions. Baseline characteristics and adverse clinical outcomes stratified by PC quintiles were compared with χ^2^-test for categorical variables and 1-way analysis of variance or the Kruskal-Wallis test for continuous variables. PC as a continuous variable was divided into quintiles in accordance with previous studies (4, 6). We took the quintile with a lowest incidence of endpoints as a reference and performed logistic regression models to explore the correlation of baseline PC with functional outcomes, and multivariable Cox regression models for its correlation with recurrent stroke and all-cause mortality. Both continuous and categorical variables that may interact with adverse clinical outcomes or PC level based on theoretical considerations or baseline characteristics were adjusted in the multivariable models. Adjusted hazard ratios (HR) or odds ratios (OR) and their 95% confidence intervals (CIs) were calculated. We used three adjusted models. Only age, gender and body mass index (BMI) were adjusted in the first model, age, gender, BMI, myocardial infarction, atrial fibrillation or flutter, drinking, smoking, hypertension, medication history of antihypertensive drugs in the second model, and all the potential confounders in the third model. In addition, we further evaluated the pattern and magnitude of correlations between PC on a continuous scale and risk of stroke recurrence, mortality and poor functional outcome using a logistic regression model or multivariable Cox regression model with restricted cubic splines for PC adjusting for covariates.

All statistical analyses were conducted with SAS software version 9.4 (SAS Institute Inc., Cary, NC). Two-tailed *P* < 0.05 were considered significantly different.

## Results

### Baseline Characteristics

Data of 16,842 subjects were eligible for statistical analysis among all 2,5018 patients in registry. Of all the involved subjects in the present study, the average age was 64.7 ± 11.9, 15274 (90.7%) had ischemic stroke, and 1568 (9.3%) had TIA. The median PC was 199 (interquartile range 165–238) × 10^9^/L. Baseline characteristics stratified into quintiles according to baseline PC were shown in Table 1. PC as a continuous variable was divided into quintiles: Q1:100–155 × 10^9^/L, (*n* = 3,296); Q2:156–185 × 10^9^/L, (*n* = 3,411); Q3:185–212 × 10^9^/L, (*n* = 3,348); Q4: 213–248 × 10^9^/L, (*n* = 3,411); Q5:249–450 × 10^9^/L, (*n* = 3,376). Compared with the rest quintiles, patients in the lowest quintile of PC were more aged and more likely to be with myocardial infarction history, atrial fibrillation and higher NIHSS score on admission. On the other hand, participants in the top quintile had higher hsCRP, and had more patients with hypertension and less patients with drinking, smoking. Patients at both ends of PC spectrum had higher WBC, CRP level, higher mRS score at discharge, and more antihypertensive drugs in hospital.

**Table 1:** Baseline characteristics of subjects stratified according to quintiles of baseline platelet count.

**Characteristics**	**Total patients**	**Platelet count quintiles, 10**^**9**^**/L**					***P*-value**
		**Q1 (100–155) (*n* = 3,296)**	**Q2 (156–185) (*n* = 3,411)**	**Q3 (185–212) (*n* = 3,348)**	**Q4 (213–248) (*n* = 3,411)**	**Q5 (249–450) (*n* = 3,376)**	
Age (SD), y	64.7 (11.9)	67.7 (11.4)	65.8 (11.7)	64.1 (11.6)	63.1 (11.9)	62.6 (12.0)	<0.001
Male, *n* (%)	10,617 (63.0)	2,264 (68.7)	2,352 (69.0)	2,171 (64.8)	2,076 (60.9)	1,754 (52.0)	<0.001
BMI (*SD*)	24.1 (3.6)	23.8 (3.5)	24.0 (3.5)	24.2 (3.6)	24.4 (3.7)	24.3 (3.7)	<0.001
**Risk factors**, ***n*** **(%)**							
Previous ischemic stroke	5,144 (30.0)	1,003 (30.4)	1,025 (30.1)	992 (29.6)	1,038 (30.4)	1,086 (32.2)	0.203
Previous myocardial infarction	406 (2.4)	97 (2.9)	98 (2.9)	76 (2.3)	72 (2.1)	63 (1.9)	0.012
Hypertension	10,720 (63.7)	2,024 (61.4)	2,134 (62.6)	2,123 (63.4)	2,224 (65.2)	2,215 (65.6)	0.001
Diabetes mellitus	3,345 (19.9)	674 (20.5)	646 (18.9)	676 (20.2)	687 (20.1)	662 (19.6)	0.546
Dyslipidemia	1,923 (11.4)	339 (10.3)	385 (11.3)	385 (11.5)	403 (11.8)	411 (12.2)	0.153
Known atrial fibrillation	1,139 (6.8)	320 (9.7)	265 (7.8)	204 (6.1)	191 (5.6)	159 (4.7)	<0.001
Drinking	4,679 (27.8)	927 (28.1)	1,042 (30.6)	946 (28.3)	967 (28.4)	797 (23.6)	<0.001
Previous or current smoker, (*n*%)	7,320 (43.8)	1,488 (45.2)	1,605 (47.1)	1,493 (44.6)	1,497 (43.9)	1297 (38.4)	<0.001
**Pre-hospital medication history**							
Antiplatelet drugs	3,290 (19.5)	679 (20.6)	671 (19.7)	601 (18.0)	677 (19.9)	662 (19.6)	0.092
Antihypertensive drugs	7,430 (44.1)	1,430 (43.4)	1,454 (42.6)	1,446 (43.2)	1,581 (46.4)	1,519 (45.0)	0.012
Lipid-lowering drug	1,149 (6.8)	222 (6.7)	228 (6.7)	220 (6.6)	243 (7.1)	236 (7.0)	0.892
Hypoglycemic drugs	2,587 (15.4)	525 (15.9)	506 (14.8)	522 (15.6)	538 (15.8)	496 (14.7)	0.511
**Index event**, ***n*** **(%)**							
Ischemic stroke	15,274 (90.7)	2,995 (90.9)	3,084 (90.4)	3,016 (90.1)	3,107 (91.1)	3,072 (91.0)	0.574
TIA	1,568 (9.3)	301 (9.1)	327 (9.6)	332 (9.9)	304 (8.9)	304 (9.0)	
NIHSS score on admission, median (IQR)	3 (1–6)	4 (1–7)	3 (1–6)	3 (1–6)	3 (1–6)	3 (1–6)	0.003
Median time from symptom onset to admission, hours		18.7 (4.5–50.0)	21.0 (5.0–51.6)	21.9 (5.5–55.0)	22.5 (5.9–56.8)	23.0 (5.3–57.4)	0.022
Intravenous thrombolysis	356 (2.1)	71 (2.2)	71 (2.1)	74 (2.2)	86 (2.5)	54 (1.6)	0.123
**Laboratory test results**							
ALT, U/L, median (IQR)	18.0 (13.0–26.2)	18.0 (13.0–26.4)	18.0 (13–16)	18.7 (13–16)	19 (13–16)	19 (13–16)	0.280
WBC,10^9^/L, median (IQR)	6.72 (5.52–8.3)	6.0 (4.9–7.42)	6.3 (5.3–7.8)	5.7 (5.6–8.1)	7.0 (5.9–8.5)	7.6 (6.3–9.4)	<0.001
CRP, mg/L, median (IQR)	4.0 (1.8–7.4)	4.3 (1.7–8.0)	4.0 (1.8–7.8)	3.6 (1.5–6.4)	4.1 (1.8–7.0)	4.6 (2.0–7.7)	0.002
hsCRP, mg/L, median (IQR)	2.5 (0.9–5.8)	2.4 (1.0–5.9)	2.3 (0.9–5.5)	2.5 (0.9–5.7)	2.3 (0.8–5.2)	2.8 (1.0–6.4)	0.040
**mRS score at discharge**							
0–2	13,060 (78.4)	2,510 (77.3)	2,654 (78.6)	2,663 (80.3)	2,679 (79.4)	2,554 (76.3)	<0.001
3–5	3,597 (21.6)	736 (22.7)	721 (21.4)	652 (19.7)	695 (20.6)	793 (23.7)	
**Medication in hospital**							
Antiplatelet drugs	14,975 (93.7)	2,864 (92.8)	3,036 (93.9)	3,012 (94.1)	3,072 (94.0)	2,991 (93.5)	0.165
Antihypertensive drugs	8,155 (67.0)	1,574 (68.1)	1,631 (66.5)	1,563 (64.4)	1,677 (67.6)	1,710 (68.1)	0.032
Lipid-lowering drugs	6,712 (92.3)	1,095 (91.2)	1,229 (92.6)	1,386 (92.3)	1,488 (92.9)	1,451 (92.1)	0.488
Hypoglycemia drug	2,864 (17.8)	565 (18.2)	571 (17.5)	596 (18.5)	590 (18.0)	542 (17.0)	0.537

*BMI, body mass index; TIA, transient ischemic attack; ALT, alanine aminotransferase; mRS, modified Rankin Scale score; NIHSS, National Institutes of Health Stroke Scale; WBC, white blood cells; IQR, interquartile range; Q, quintile*.

### Association of PC With Adverse Clinical Outcomes

**Figure 1:**
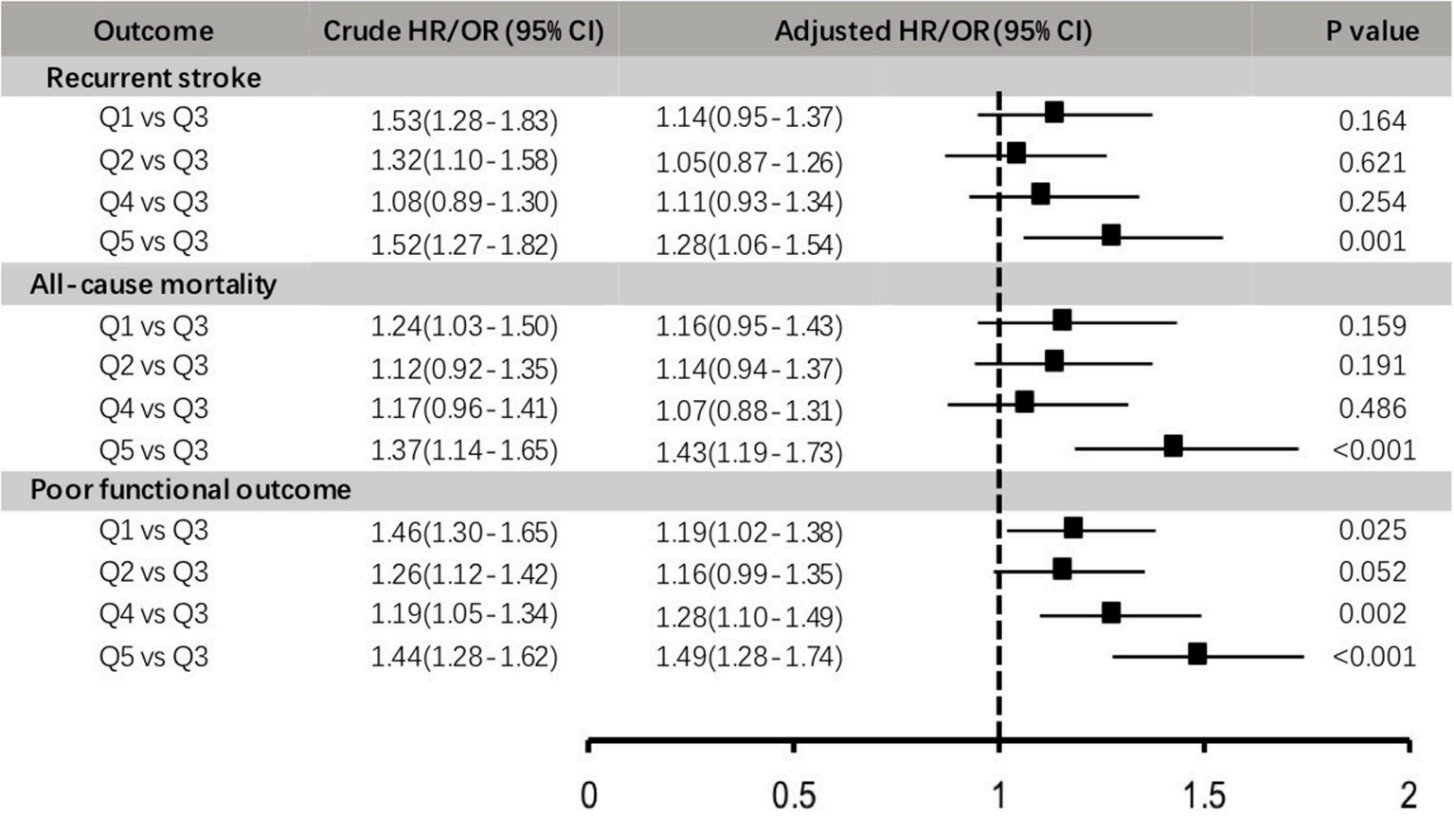
Crude and adjusted hazard ratios (HR)/odds ratios (OR) for 1-year adverse clinical outcomes stratified by platelet count levels.

After 1-year follow-up, 1241 (7.4%) recurrent stroke occurred, 3,557 (21.1%) subjects ended up with poor functional outcome, and 1,377 (8.2%) subjects died. The risk of adverse clinical outcomes in quintiles were shown in Table 2 and Figure 1 in crude and adjusted odds ratios with 95% confidence intervals (95% CI).

**Table 2:** Risk of recurrent stroke, all-cause mortality, and poor functional outcome stratified by platelet count levels at 1-year follow-up.

**Outcomes**	**Quintile of platelet count, × 10^**9**^/L**	**Events *n* (%)**	**Model 1^a^**	***P*-value**	**Model 2^b^**	***P*-value**	**Model 3^c^**	***P*-value**
			**Adjusted HR^d^/OR^e^ (95% CI)**		**Adjusted HR^d^/OR^e^ (95% CI)**		**Adjusted HR^d^/OR^e^ (95% CI)**	
Recurrent stroke	Q1, 100–155	255 (7.7)	1.13 (0.94–1.35)	0.199	1.10 (0.92–1.32)	0.306	1.14 (0.95–1.37)	0.164
	Q2, 156–185	239 (7.0)	1.06 (0.88–1.28)	0.522	1.04 (0.87–1.26)	0.646	1.05 (0.87–1.26)	0.621
	Q3, 186–212	212 (6.3)	1		1		1	
	Q4, 213–248	249 (7.3)	1.18 (0.98–1.42)	0.076	1.18 (0.98–1.42)	0.074	1.11 (0.93–1.34)	0.254
	Q5, 249–450	286 (8.5)	1.39 (1.17–1.67)	<0.001	1.40 (1.17–1.67)	<0.001	1.28 (1.06–1.54)	0.001
All-cause mortality	Q1, 100–155	316 (9.6)	1.18 (0.99–1.40)	0.065	1.14 (0.96–1.35)	0.145	1.16 (0.95–1.43)	0.159
	Q2, 156–185	285 (8.4)	1.13 (0.95–1.35)	0.171	1.10 (0.92–1.32)	0.279	1.14 (0.94–1.37)	0.191
	Q3, 186–212	217 (6.5)	1		1		1	
	Q4, 213–248	237 (7.0)	1.12 (0.93–1.35)	0.227	1.13 (0.94–1.36)	0.189	1.07 (0.88–1.31)	0.486
	Q5, 249–450	322 (9.5)	1.62 (1.36–1.93)	<0.001	1.66 (1.39–1.97)	<0.001	1.43 (1.19–1.73)	<0.001
Poor functional outcome	Q1, 100–155	780 (23.7)	1.19 (1.05–1.35)	0.007	1.17 (1.03–1.33)	0.017	1.19 (1.02–1.38)	0.025
	Q2, 156–185	718 (21.1)	1.14 (1.01–1.30)	0.040	1.12 (0.99–1.28)	0.073	1.16 (0.99–1.35)	0.052
	Q3, 186–212	585 (17.5)	1		1		1	
	Q4, 213–248	685 (20.1)	1.27 (1.12–1.44)	<0.001	1.27 (1.12–1.45)	<0.001	1.28 (1.10–1.49)	0.002
	Q5, 249–450	789 (23.4)	1.61 (1.42–1.83)	<0.001	1.63 (1.44–1.85)	<0.001	1.49 (1.28–1.74)	<0.001

*OR, odds ratio; HR, hazard ratio; CI, confidence interval; Q, quintile*.

a *Model 1: adjusted for age, gender and BMI*.

b *Model 2: adjusted for age, gender, BMI, myocardial infarction, atrial fibrillation or flutter, drinking, smoking, hypertension, medication history of antihypertensive drugs*.

c *Model 3: adjusted for age, gender, BMI, myocardial infarction, atrial fibrillation or flutter, drinking, smoking, hypertension, medication history of antihypertensive drugs, NIHSS score on admission, white blood cell, and mRS score at discharge*.

d *Adjusted HR for recurrent stroke and all-cause mortality*.

e *Adjusted OR for poor functional outcome*.

Compared with the third quintile of PC (186–212 × 10^9^/L) taken as reference, patients in the top quintile (249–450 × 10^9^/L) were significantly associated with adverse clinical outcomes (*p* for trend < 0.05), including recurrent stroke, all-cause mortality and poor functional outcome (mRS 3–6), the adjusted HR/OR was 1.28 (1.06–1.54), 1.43 (1.19–1.73), and 1.49 (1.28–1.74), respectively. In addition, the lowest group of PC (100–155 × 10^9^/L) was associated with an increased risk of poor functional outcome (adjusted OR 1.19, 95%CI 1.02–1.38) when comparing with reference group, and with a trend toward higher risk of recurrent stroke and mortality although not statistically significant (adjusted HR 1.14 [0.95–1.37], *p* = 0.164; adjusted HR 1.16[0.95–1.43], *p* = 0.159, respectively).

**Figure 2:**
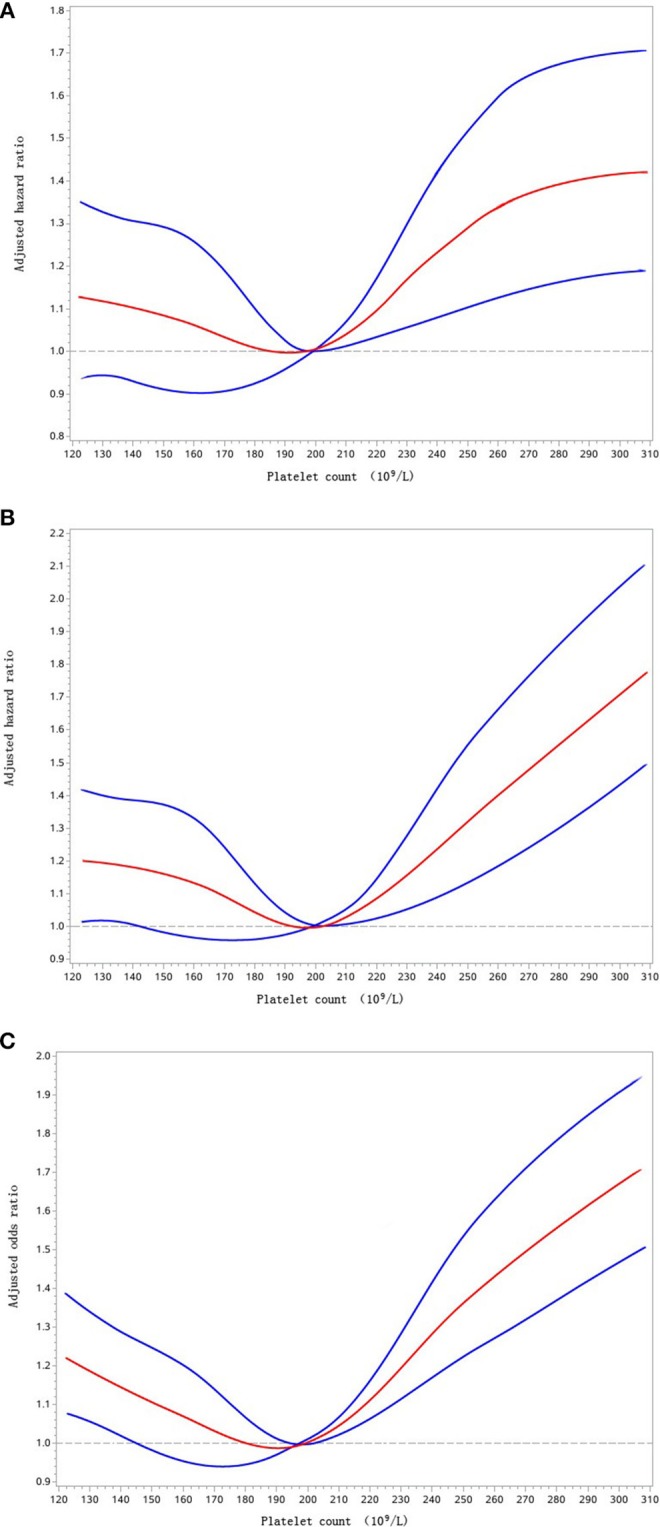
Adjusted hazard ratios of recurrent stroke **(A)**, all-cause mortality **(B)**, and adjusted odds ratio of poor functional outcome **(C)** according to platelet count. Red lines indicate adjusted hazard/odds ratio, and blue lines indicate the 95% confidence interval bands. Reference is platelet count of 199 × 10^9^/L. Data were fitted with a Cox regression model of restricted cubic spline with 5 knots (5th, 25th, 50th, 75th, and 95th percentiles) for platelet count, with adjustment for potential covariates. The lowest 5% and highest 5% of participants are not shown for small sample sizes.

Further cox/logistic regression analyses with restricted cubic spline indicated that higher baseline PC levels significantly associated with elevated risk of recurrent stroke (Figure 2A) and all-cause mortality (Figure 2B), both higher and lower baseline PC levels were significantly correlated with an increased risk of poor functional outcome (Figure 2C).

## Discussion

Subanalysis of this large sample prospective registry study suggested a J-shaped relationship between baseline PC and recurrent stroke/all-cause mortality, and a U-shaped relationship between baseline PC and long-term poor functional outcome in ischemic stroke or TIA patients within PC 100–450 × 10^9^/L. This shall be further interpreted as that both higher (213–248 × 10^9^/L, 249–450 × 10^9^/L) and lower (100–155 × 10^9^/L) circulating PC levels were significantly correlated with increased risk of poor functional outcome compared with intermediate reference PC level (186~212 × 10^9^/L). Moreover, higher baseline PC (249–450 × 10^9^/L) was also associated with increased risk of recurrent stroke and mortality. Thus, the low cost and universal availability of laboratory testing for PC would make PC level an alternative prognostic biomarker for ischemic stroke or TIA patients with normal platelet count levels, although further prospective studies are essential.

We believe that this is the largest prospective study to date investigating the correlation of baseline PC and clinical outcomes in ischemic stroke or TIA patients. Unique for our analysis, we limited the subjects within normal PC range (100–450 × 10^9^/L) according to international guidelines (10). In order to guarantee that the abnormal PC by itself didn't lead to differences on healthcare service provided and frequency of disease detected, patients with thrombocytopenia (PC < 100 × 10^9^/L) and thrombocytosis (PC > 450 × 10^9^/L) were excluded for its established correlations with poor clinical outcomes in both ischemic cerebrovascular and cardiovascular disease in our study (11, 12). Therefore, the platelet count interval we chose increased the internal validity of the study. In addition, our results were not completely consistent with some previous results that demonstrated a strong correlation between lower PC and mortality both in cardiovascular and cerebrovascular disease (4, 12). We speculated that the exclusion of patients with PC < 100 × 10^9^/L (*n* = 542), which has been confirmed as an independent predictor of mortality (4, 12), might account for this discrepancy, although more confirmative studies are needed. In summary, it suggested that patients at both ends of normal PC spectrum bore higher risk of adverse clinical outcome compared with intermediate PC level.

Circulating platelets play critical role in the development, progression, and resolution of ischemic stroke, not only due to their direct effects upon the endothelium but also by acting as mediator for other circulating cells through facilitating activation and discharging of activators stored in platelet granules (13). Some studies indicated that PC was significantly lower in patients with ischemic stroke and myocardial infarction compared with healthy controls (14, 15). Researchers speculated the pre-thrombotic state derived from increasing consumption of platelets in the process of platelet activation and subsequent atherothrombosis accounted for the decreases in PC. However, non-significant even contradictory (4, 16) correlations between PC and clinical outcomes of cardio-cerebrovascular diseases were presented in other studies. Mueller et al. (4) conducted a prospective observational study included 1,616 consecutive unstable angina/non-ST-segment elevation myocardial infarction patients and found a non-linear and U-shaped association between PC and long-term mortality. Recently Du et al. (16) found a positive correlation between elevated PC level and the risk of stroke recurrence, but without significant association with poor prognosis. The small sample size, heterogeneous inclusion and grouping criteria of these studies partly accounted for the controversial results. Moreover, no relevant studies were reported for now on ischemic stroke or TIA patients with normal PC levels.

Our finding can be explained mainly by two mechanisms: platelet consumption and platelet-induced inflammation during platelet activation. Specifically, patients in the lower quintile (100–155 × 10^9^/L) were older and undertook heavier atherosclerosis burden for cerebrovascular disease compared with other quintiles, including previous myocardial infarction, atrial fibrillation, drinking, and smoking (Table 1). As atherosclerosis progressed, it implied more platelet activation and atherothrombosis derived from increased formation of leukocyte-platelet aggregation (WBC-platelet complex) (17), which may enhance recruitment of leukocytes/macrophages, aggregation and consumption of platelets (18). The significantly lower level of WBC in lower quintiles partially verified this WBC-platelet complex hypothesis in stages of atherogenesis.

On the other hand, the finding of more adverse clinical outcomes for patients with PC ≥ 249 × 10^9^/L may be attributed to another reason. Some studies have presented a positive correlation between PC level and platelet-induced pro-thrombotic or pro-inflammatory mediators, such as soluble CD40 ligand (sCD40L) (19), RANTES chemokine (20) and thromboxane A_2_. sCD40L is one of the most studied transmembrane protein induced by activated platelets. Viallard et al. (19) has demonstrated a tight correlation between circulating PC level and serum sCD40L that PC level increased in both essential thrombocythemia and reactive thrombocytosis. These results can be interpreted as that highly reactive and numerous platelets represent great risk for pro-thrombotic or pro-inflammatory states. Regrettably, the CNSR II study did not collected whole information about sCD40L and other platelet-associated pro-thrombotic mediators. However, higher levels of baseline CRP and hsCRP in the top PC quintile, another two verified predictors of adverse clinical outcome (21, 22), confirmed its hyper-inflammatory state from another aspect. In general, we can possible speculate that the two mechanisms, platelet consumption and platelet-induced inflammation, worked together, interacted with each other, and reached a relatively lower activity at intermediate PC level, making platelet count can be of prognostic significance for improved risk stratification of adverse clinical outcomes in ischemic stroke and TIA patients, although more fundamental researches are essential to explain this.

The main strength of this study lied in the large, multicentric, consecutive patient inclusion design. All demographic, clinical, and follow-up data were prospectively collected and supervised, allowing subanalysis adjusted for potential confounders. However, several limitations should not be ignored. First, our study included stroke or TIA patients within 7 days after symptom onset instead of within hyperacute 24 h, and platelet count was collected only at baseline instead of longitudinal data. Although platelet indexes are relatively stable, some studies reported the influences of alcohol, antiplatelet agents, and lipid-lowing drugs on changes of platelet indexes (23, 24). Second, baseline platelet count testing was conducted at each center. Measurement errors stemmed from different analytic systems cannot be completely excluded. However, all platelet indexes results should be comparable because platelet count measurement in each center were performed according to recommendation of International Federation of Clinical Chemistry and Laboratory Medicine (2011). Finally, residual confounding factors from comorbidities or environment may still exist and influence circulating platelet indexes, such as tumor, blood disease, and acute toxicosis (25, 26).

## Conclusion

In conclusion, we found a J-shaped relation between baseline platelet count and higher risk of stroke recurrence/all-cause mortality, a U-shaped relation between baseline platelet count and poor functional outcomes in ischemic stroke or TIA patients with normal platelet count. The low cost and universal availability of laboratory testing for platelet indexes would make platelet count level an alternative prognostic biomarker for ischemic stroke or TIA patients with normal platelet count, although further research is essential.

## Author Contributions

This work was conceptualized by MY, YoW, and YiW. All authors approved the protocol. Data collection was done by ZL, XZ, LL, JJ, and XM. Statistical analysis was undertaken by YP, HY, and MY. MY, ZL, and JJ prepared the manuscript. YiW and YoW are the guarantors of this paper.

### Conflict of Interest Statement

The authors declare that the research was conducted in the absence of any commercial or financial relationships that could be construed as a potential conflict of interest.
